# Liberty or life: mental health care in Australia

**DOI:** 10.1017/S1092852924000634

**Published:** 2024-10-31

**Authors:** Kirsty MacDonald, Andrew Ellis

**Affiliations:** 1 Psychiatrist Justice Health NSW, UNSW, Kensington, Australia; 2 Clinical Director Justice Health NSW, UNSW, Kensington, Australia

**Keywords:** Mental health care in Australia, mental health, Australia, liberty or life, mental health care

## Abstract

This article reviews the development of mental health and psychiatric services in Australia for the international reader. The development of relevant legislation, health-care systems, and the effectiveness of treatment for people with schizophrenia is reviewed. Gaps in service delivery and future directions are considered.

## Introduction

The indigenous inhabitants of the Australian continent arrived approximately 65 000 years ago. Treatment for schizophrenia prior to European arrival is not well known by current professionals. The Commonwealth of Australia, a parliamentary democracy, was established in 1901. The Commonwealth is a federation of six states and two territories that were originally colonies of Britain. Australia has a population of 26.8 million people, 30% of whom are born overseas. Indigenous Australians make up 3.8% of the population. Schizophrenia is a complex disorder of brain functioning, which the World Health Organization describes as a “disturbance involving the most basic functions that give the normal person a feeling of individuality, uniqueness and self-direction.”^1^ Schizophrenia affects up to 1% of the population^2^ and is among the top 10 disorders in the global burden of disease and disability.^3^ Australian surveys show similar prevalence results to international studies.^4^

Treatment of schizophrenia includes staging of the disorder, psychological interventions (such as Cognitive Behavioral Therapy), addressing co-morbid conditions such as substance use and interpersonal deficits, psychoeducation, and antipsychotic medications.^5^ Treatment guidelines detail the management of acute and chronic psychotic symptoms, as well as ongoing evaluation of efficacy, adherence, and addressing co-morbid issues.^5^ Indigenous Australians show higher rates of schizophrenia, which may be explained by higher rates of substance use^6^ disorders, although other elements such as poverty, racism, and inequitable access to health care also play a role.^6^

## Overview of legislation

In Australia, individual states have their own Mental Health Acts to guide decision-making around involuntary treatment and admission. Each piece of legislation falls under the jurisdiction of the State or Territory government. Mental Health Acts guide admission of all civil involuntary admissions, based on current (and future) mental state and risk of harm to self or others.^7^ Currently, variations between the definition of mental illnesses and disorders and differing criteria for involuntary treatment are found within the Acts.^8^

The below table details current legislation for the states and territories in Australia for involuntary commitment and treatment.^8^

**Table 1:** 

	ACTMental Health Act 2015s58,66 101	NSWMental Health Act 2007ss12,14,68	NTMental Health and Related Services Act 1998s14	QLDMental Health Act 2016ss3, 12	SAMental Health Act 2009s21	TASMental Health Act 2013ss6, 40	VICMental Health and Wellbeing Act 2022s89, 142, 143	WAMental Health Act 2014s25
Mental illness	The person has a mental illness or mental disorder.	The person is suffering from mental illness and, owing to that illness, there are reasonable grounds for believing that the care, treatment, and control of the person is necessary.	The person has a mental illness and as a result of the mental illness, without treatment the person is likely to:	The person has a mental illness; because of the person’s illness, the absence of involuntary treatment, or the absence of continued involuntary treatment, is likely to result in:	The person has a mental illness and because of the mental illness, the person requires treatment for	The person has, or appears to have, a mental illness and without treatment, the mental illness will, or is likely to, seriously harm:	The person has a mental illness; and because the person appears to have a mental illness, the person appears to need immediate treatment to prevent:	The person has a mental illness for which the person is in need of treatment and because of the mental illness, there is:
Harm	Is doing, or is likely to do, serious harm to themselves or someone else.	For the person’s own protection from serious harm or the protection of others from serious harm.	Cause serious harm to himself or herself or to someone else	Imminent serious harm to the person or others or	The person’s own protection from harm (whether physical or mental and including harm involved in the continuation/deterioration of the person’s condition) or to protect others from harm and	The safety of the person or others or	Serious harm to the person or to another person	A significant risk to the safety of the person or another, or a significant risk of serious harm to the person or to another or
Need for care	Is suffering, or is likely to suffer, serious mental or physical deterioration.	N/A	Suffer serious mental or physical deterioration and	The person suffering serious mental or physical deterioration.	The person has impaired decision-making capacity relating to appropriate treatment of the person’s mental illness;	The person’s health and	Serious deterioration in the person’s mental or physical health	A significant risk to the health of the person and
Psychiatric treatment	Treatment/care/support is likely to reduce the harm or deterioration (or its likelihood) or result in an improvement in the person’s condition.	N/A	The person requires treatment that is available at an approved treatment facility and	N/A	N/A	The treatment will be appropriate and effective in terms of the outcomes referred to in section 6(1) [see additional criteria] and	If the person is made subject to a temporary treatment order or treatment order, the immediate treatment will be provided to them; and	Treatment in the community cannot reasonably be provided to the person and
No less restrictive alternative	The treatment, care, or support cannot be adequately provided in another way that would involve less restriction of the freedom of choice and movement.	No other care of a less restrictive kind that is consistent with safe and effective care, is appropriate and reasonably available to the person	There is no less restrictive means of ensuring that the person receives the treatment and	The main objects of the Act are to be achieved in a way that is the least restrictive of the rights and liberties of a person who has a mental illness.	There is no less restrictive means than an inpatient treatment order (ITO) to ensure appropriate treatment of the person’s illness.	The treatment cannot be adequately given except under a treatment order.	There are no less restrictive means reasonably available to enable the person to receive the immediate treatment.	The person cannot be adequately provided with treatment in a way that would involve less restriction.
Additional criteria	The above criteria must be satisfied before a mental health order can be made for a person with decision-making capacity (DMC) who refuses treatment, care, or support; the harm or deterioration must be so serious that it outweighs the right to refuse. If a person lacks DMC and refuses treatment, care of support, the only criteria that apply is the existence of a mental disorder or illness.	In considering whether a person is a mentally ill person, the continuing condition of the person, including any likely deterioration in the person’s condition, and the likely effect of any such deterioration, are to be taken into account.	The person is not capable of giving informed consent to the treatment or has unreasonably refused to consent to the treatment.	The person does not have consent to be treated for the illness.	In considering whether there is no less restrictive means than an ITO of ensuring appropriate treatment, consideration must be given, amongst other things, to the prospects of the person receiving all necessary treatment on a voluntary basis or in compliance with a community treatment order.	(i) The person does not have DMC (ii) the treatment will: prevent/remedy mental illness; or manage/alleviate it where possible; or reduce the risks that persons with mental illness may pose to themselves or others; or monitor and evaluate the person’s mental state	(i) the person does not have the capacity to give informed consent.	(i) The person does not demonstrate the capacity to make a treatment decision about the provision of treatment; (ii) decisions regarding ICT must be made with reference to guidelines published by the Chief Psychiatrist

All states and territories include a similar definition of “mental illness”; however, only the Australian Capital Territory (ACT) and New South Wales (NSW) have a definition for “mental disorder.” Other differences include a “continuing condition” in NSW, which includes potential deterioration or likely deterioration in their care. South Australia (SA) requires a person to have impaired decision-making capacity. As mentioned by Tosson et al.,^9^ “Criteria for mental health treatment is too diversely defined in each jurisdiction. While the criteria adhere to the ethical principles of beneficence and non-maleficence, they vary widely in implementation, which may result in differing treatments between States and Territories.”

Mental Health Acts are also used to determine acute treatment in inpatient settings and involuntary longer-term treatment in the community, under the provision of compulsory Community Treatment Orders (CTOs). Research of the reporting process of involuntary treatment both in hospitals and in the community is different among jurisdictions and uses differing data (incidents of treatment versus number of individuals affected).^10^ There are clear differences regarding the reporting of involuntary treatment across states and territories within Australia and this should be rectified in order to inform current and future practice.

Australia is signatory to the Convention on the Rights of Persons with Disabilities (CRPD) and the Optional Protocol to the Convention against Torture and other Cruel, Inhuman or Degrading Treatment or Punishment (OPCAT). The Australian Government interprets the CRPD as allowing for “compulsory assistance or treatment of persons, including measures taken for the treatment of mental disability, where such treatment is necessary, as a last resort and subject to safeguards.” Although signatory to OPCAT, Australia has refused entry to international regulatory observers to review facilities where persons are detained (prisons, psychiatric hospitals, and locked community homes).^11^

There are forensic provisions for diversion of persons with mental illness charged with criminal offenses into health systems, with each state administering services in varying fashion.^12^ This includes persons who meet legal criteria for not having criminal responsibility for their acts, not being capable of performing trial tasks, or for prisoners who require involuntary treatment of their condition. Lower order offences may be dealt with summarily by diversion to mental health treatment.^13^ These provisions are not consistently applied across the country,^14^ and persons who may be eligible for diversion can remain in prison settings which is not recommended or effective.

## Services for people with schizophrenia

One of the unique health-care differences within Australia is the split between Commonwealth and state funding for health services. The Commonwealth government oversees the broad delivery of health care and funds Medicare (a universal safety net for outpatient care), whereas the individual states and territories are responsible for their hospital care and budget. This impacts the treatment of a chronic and complex illness like schizophrenia, which requires coordinated inpatient and outpatient services for integrated management.

The National Mental Health Service Planning Framework (NMHSPF)^15^ is one model that the Commonwealth government has introduced which assists with providing services for their local community. The broad principles are (i) mental health promotion, (ii) mental illness prevention, (iii) primary and specialized clinical ambulatory mental health services, (iv) specialized mental health community support services, (v) specialized bed-based mental health-care services, and (vi) medications and procedures.

Different jurisdictions within Australia broadly offer models of care that include inpatient treatment, community, and outreach treatment. The specific needs of an individual should guide treatment.^5^ Typically, patients needing acute admissions (due to symptoms and risk) might be treated in an inpatient ward. After some time, they might transition into community care. Some will be managed with community treatment orders (CTOs), as per local legislation and policies.

From a treatment perspective, there has been criticism that the legislative requirements requiring patients to be a danger to others or themselves delay treatment and lead to worsened outcomes.^16^ This is because delays in treatment ultimately lead to a longer duration of untreated psychosis, which might be linked to both suicide^17^ ^–^^19^ and violence risk^20^ ^,^^21^ as well as worsen the prognosis of the illness itself.^22^ ^,^^23^ The ethical issues of autonomy and beneficence are raised in this setting, which are keenly monitored in both medical and legislative frameworks.

The current system of having patients present to their local emergency departments for assessment also places pressure on the departments themselves. Poor access to community care flows into increased pressure being placed on emergency departments when dealing with acute presentations of mentally unwell individuals. Poor planning, coordination, and accountability mechanisms need to be addressed to improve equitable access for people who present with mental health concerns to their local hospital. As highlighted by emergency clinicians, the mental health system is ‘highly fragmented, with unclear roles and responsibilities’.^24^

## Effectiveness of treatment

Funding for mental health conditions across the Commonwealth and state/territories varies. Additionally, funding addresses both high- and low-prevalence disorders, as well as preventive strategies.^25^ It is difficult to find the overall money spent on treatment of schizophrenia within the Australian context. The term “mental health” encompasses many disorders, as well as prevention strategies. This may lead to legislative and service reforms that reflect advocacy from groups representing high-prevalence conditions and neglect the special concerns faced by persons with schizophrenia and their families.

Treatment of mental health conditions should always be individualized and catered to the individual. Individuals suffering from more common disorders such as anxiety and depression tend to have greater insight and adherence to treatment than people suffering from schizophrenia. Management plans that address higher prevalent conditions might not adequately meet the treatment needs of people suffering from schizophrenia.

Treatment measures such as CTOs are often used to manage complex clients with schizophrenia in the community. However, CTOs should not be purely used to gain access to services, nor are they effective when services are non-existent or inadequate.^26^ Research also highlights that people with culturally and linguistically diverse backgrounds – including Indigenous Australians – are more likely to be placed on compulsory community treatment.^27^

Small studies that have evaluated the experience of people suffering from schizophrenia highlight universal goals – including having a stable place to live, remaining independent, and keeping physically healthy. Additionally, having autonomy and being able to collaborate with their treating team was very important.^28^

## Conclusion

Long-term management of complex conditions such as schizophrenia requires individualized and highly specialized care. Current models of care and associated funding arrangements by both Commonwealth and state/territory governments do not adequately address the needs of this vulnerable community. Specialized models of care for people with severe mental illnesses would ensure that breakdowns in treatment provision were minimized and crisis presentations were not the mainstay of obtaining care within the public health system. Schizophrenia is often a forgotten disorder, where the lack of advocacy leads to poorer outcomes – for both the individual and our society.

Overall, the legislative regimes and health systems provided for persons with schizophrenia show confusion, perhaps reflecting the disorganization and lack of insight characteristic of the condition itself. It has been long noted that people without schizophrenia find the condition difficult to understand.^29^ On the one hand, legislation for compulsory treatment of mental disorders in general places schizophrenia as no different from other forms of mental disorder and distress and thereby makes compulsory hospitalization difficult to achieve, placing greater weight on autonomy and personal choice around treatments. On the other, hurdles to achieve diversion from justice systems are also high. When involuntary treatment or imprisonment does occur, international scrutiny of practice is then reduced. Services developed around these seemingly divergent objectives can then fail to develop and provide services relevant to the minority of mental health patients with the arguably more severe condition of schizophrenia. The end result is high rates of persons with schizophrenia in prisons,^30^ high and increasing rates of homelessness,^31^ and mortality.^32^
